# N-Acetylcysteine Prevents Arsenic-Induced Apoptosis but Not Supernumerary Motor Neuron Development in Zebrafish Embryos: Assessment of Protein Carbonylation and the p53 Pathway

**DOI:** 10.3390/ijms27073263

**Published:** 2026-04-03

**Authors:** Qiang Gu, Camila S. Silva, Nathan C. Twaddle, Frederick A. Beland, Jyotshna Kanungo

**Affiliations:** 1 Division of Neurotoxicology, National Center for Toxicological Research, U.S. Food and Drug Administration, 3900 NCTR Road, Jefferson, AR 72079, USA; 2 Division of Biochemical Toxicology, National Center for Toxicological Research, U.S. Food and Drug Administration, Jefferson, AR 72079, USA

**Keywords:** arsenic, protein carbonylation, apoptosis, motor neurons, reactive oxygen species, zebrafish

## Abstract

Arsenic induces apoptosis in both cancerous and non-cancerous cells. The mechanism of arsenic-induced apoptosis is complex. We previously demonstrated that the antioxidant acetyl L-carnitine prevented sodium arsenite-induced apoptosis in zebrafish embryos. To gain more insight into the mechanism of arsenic-induced apoptosis, we explored the effect of another antioxidant, N-acetylcysteine (NAC). Co-treatment of sodium arsenite with 1 or 2 mM NAC had no effect on zebrafish development. There was a significant but partial reduction in apoptosis in the embryos co-treated with sodium arsenite and 1 mM NAC, while embryos treated with 1 mM NAC alone showed the loss of normal apoptosis that was observed in the control embryos. Complete abolition of apoptosis occurred in embryos co-treated with sodium arsenite and 2 mM NAC; however, 2 mM NAC alone resulted in 100% mortality, indicating antioxidant toxicity at high doses. NAC (1 mM) did not prevent sodium arsenite-induced increase in motor neurons, suggesting that arsenic-induced apoptosis and supernumerary motor neuron development are mediated via distinct pathways. To determine whether NAC prevented arsenic-induced apoptosis via reactive oxygen species (ROS) signaling, we assessed ROS levels and oxidative modification of proteins (carbonylation) using an OxyBlot assay. Neither sodium arsenite nor NAC altered protein oxidation, ROS levels, or p53, a pro-apoptotic protein, transcript levels. Additionally, dicoumarol, an inducer of p53 protein degradation, did not inhibit sodium arsenite-induced apoptosis. These results indicate that protein oxidation and p53 signaling are not involved in arsenic-induced apoptosis and that NAC prevents arsenic toxicity in zebrafish embryos through a hitherto unknown mechanism.

## 1. Introduction

Arsenic is a well-established environmental toxicant that poses significant public health risks globally, primarily through contaminated drinking water and food sources. The World Health Organization (WHO) has identified this metalloid as one of ten chemicals of major public health concern worldwide [1]. Human exposure to arsenic occurs through multiple routes, including consumption of contaminated water and food, contact with industrial products and pharmaceuticals, and inhalation [2,3]. The U.S. Environmental Protection Agency (EPA) has established a maximum contaminant level of 10 µg/L (10 parts per billion) for arsenic in drinking water. Chronic arsenic exposure has been linked to numerous adverse health outcomes, including increased cancer risk, neurological disorders, cardiovascular disease, and reproductive and developmental complications [4,5]. Paradoxically, while chronic arsenic exposure demonstrates carcinogenic potential in humans [5], arsenic compounds are also employed therapeutically to treat certain cancer types [6,7,8]. Arsenic induces apoptosis in various cell types, including human keratinocytes [9] and immune cells such as T-lymphocytes [10], thereby contributing to arsenicosis [11] and immune dysfunction [12]. This proapoptotic effect has been associated with cancerous skin conditions [12]. Arsenic-induced apoptosis operates through the activation or inactivation of multiple signaling cascades [13], including oxidative stress pathways and the tumor suppressor protein p53 [14,15].

Arsenic is a known inducer of reactive oxygen species (ROS) [16] that causes oxidative stress in mammalian cells (reviewed in [17]). Both arsenic trioxide and sodium arsenite directly induce ROS production through multiple mechanisms, including modification of ROS-controlling enzymes [16]. Antioxidants have demonstrated the ability to counteract the harmful effects of toxic elements, including arsenic (reviewed in [17]). Acetyl L-carnitine (ALCAR), which functions as both a voltage-gated calcium channel modulator [18] and an antioxidant [19], has been shown to mitigate arsenic-induced oxidative stress in the rat hippocampus [20] and prevent arsenic-induced apoptosis in zebrafish (*Danio rerio*) embryos [21]. In this study, we investigate whether N-acetylcysteine (NAC) similarly protects zebrafish embryos from arsenic-induced apoptosis [22]. NAC is an FDA-approved therapeutic agent for treating acetaminophen overdose in both animals and humans [23]. Additionally, in vitro studies have demonstrated that NAC functions as a calcium modulator [24,25].

NAC, a precursor to glutathione, is a powerful antioxidant that protects cells from oxidative stress-induced damage by scavenging ROS [26]. In rats, NAC has been shown to ameliorate manganese-induced neurotoxicity by diminishing behavioral deficits, normalizing acetylcholinesterase activity, and reducing ROS levels [27]. In human neuronal progenitor cells, NAC prevented arsenite-mediated induction of heme oxygenase-1 (HMOX1) expression—a protein involved in oxidative stress responses—and reduced the differentiation of neurons and oligodendrocytes [28]. Sodium arsenite is a known inducer of ROS [29].

Paradoxically, arsenic trioxide and other arsenical compounds have therapeutic applications for certain conditions, including FDA-approved treatments for multiple myeloma, promyelocytic leukemia, and other lymphomas, and can inhibit inflammasomes in mice [30]. A potential mechanism underlying arsenic’s efficacy in cancer treatment involves the induction of apoptosis and oxidative damage (reviewed in [31]). However, NAC reversed the protective effects (inflammasome inhibition) conferred by arsenical compounds (arsenic trioxide and sodium arsenite) in macrophages [30], suggesting that arsenic and NAC can counteract each other’s effects.

Multiple studies have demonstrated NAC’s protective effects against arsenic-induced cellular damage. In vitro, NAC prevented arsenic-induced endoplasmic reticulum stress in Neuro-2a cells [32] and attenuated apoptosis induced by an arsenic and dopamine mixture in the SH-SY5Y neuroblastoma cell line [33]. In mouse cerebral cortex, while arsenic induced oxidative stress and apoptosis, NAC prevented apoptotic cell death [34]. In zebrafish models, NAC ameliorated ROS generation and impaired photomotor response and thigmotaxis induced by environmentally relevant arsenic concentrations (5, 10, 50, and 100 µg/L) [35]. NAC also prevented arsenic-induced apoptosis in a zebrafish liver cell line [36]. However, one study on zebrafish embryos showed that NAC ameliorated arsenic-induced apoptosis only in a very restricted cell population in the head region [37]. Environmentally relevant arsenic concentrations (≤150 µg/L) have been shown to induce apoptosis specifically in the yolk sac of zebrafish larvae following acute exposure [38]. Despite these findings, the effects of arsenic on cell survival throughout the whole zebrafish embryo in vivo and on protein carbonylation have not been demonstrated.

Zebrafish embryos are an ideal alternative model system to study toxicities induced by drugs and chemicals [39]. The use of zebrafish in disease modeling has also been increasing over the last several decades [40,41]. Our previous studies demonstrated that inorganic arsenic alters the development of the nervous system, including dopaminergic neuron and motor neuron development, but not serotonergic neuron development in zebrafish embryos [42]. Using zebrafish embryos, which are suitable for in-depth mechanistic studies, we also showed that arsenic induced supernumerary motor neuron development through activation of the Sonic hedgehog (Shh) pathway [42]. However, the mechanism of arsenic-induced apoptosis was not revealed in those studies.

Arsenic induces apoptosis in both cancerous and healthy cells, including myoblasts, pancreatic cells, brain cells [33,43,44,45], keratinocytes [9], and T-lymphocytes [10]. One mechanism involves arsenic-induced oxidative stress [46], which causes DNA damage and subsequently activates p53, triggering apoptosis [47,48]. Studies using human gastric cancer and glioblastoma cell lines have demonstrated p53’s role in this process: when p53 was inhibited using antisense oligonucleotides, both caspase activation and apoptosis were suppressed [49,50]. Protein carbonylation, the formation of stable carbonyl groups (aldehydes/ketones) on amino acids, serves as a biomarker of oxidative stress [51].

In the current study, we used zebrafish embryos at 5 h post-fertilization (hpf), a developmental stage characterized by gastrulation and cellular differentiation. At this timepoint, embryos have passed the mid-blastula transition (3–3.5 hpf) and begun embryonic gene transcription [52], making them a more representative multicellular model than the transcriptionally quiescent one-cell stage immediately following fertilization. We investigated the mechanisms underlying arsenic-induced apoptosis in these embryos, focusing on protein carbonylation, ROS generation, and the p53 pathway. Additionally, we assessed whether N-acetylcysteine (NAC), an antioxidant, could ameliorate arsenic-induced apoptosis.

## 2. Results

### 2.1. Sodium Arsenite Prevents Mortality of Zebrafish Embryos Exposed to High Concentrations of N-Acetylcysteine (NAC)

**Figure 1:**
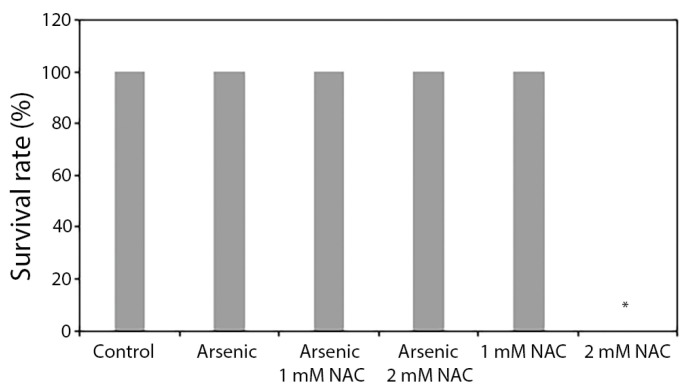
Effect of arsenic and NAC, alone or in combination, on zebrafish survival. *Hb9-GFP* transgenic zebrafish embryos at 5 hpf were exposed (static) to 200 mg/L of sodium arsenite in the presence or absence of 1 or 2 mM NAC (*n* = 10/treatment group). After 67 h of exposure (embryo age—72 hpf), survival rates were assessed. Data are presented as mean ± SEM. * Significant difference (*p* < 0.05) from the control. The absence of error bars is due to the lack of variation in data from each sample. N-acetylcysteine = NAC; sodium arsenite = Arsenic.

To assess the effects of sodium arsenite on development in the presence of NAC, 5 hpf zebrafish embryos were exposed to sodium arsenite at 200 mg/L in the presence of 1 or 2 mM NAC for 67 h. In the presence or absence of NAC, sodium arsenite did not affect zebrafish embryo survival (Figure 1). NAC at 1 mM did not affect survival; however, NAC at 2 mM alone induced 100% mortality (Figure 1). The results indicate that NAC, at a higher concentration (2 mM), can be developmentally toxic to zebrafish embryos and such toxicity is prevented in the presence of sodium arsenite.

### 2.2. NAC at 1 mM Attenuates Sodium Arsenite-Induced Apoptosis in Zebrafish Embryos

**Figure 2:**
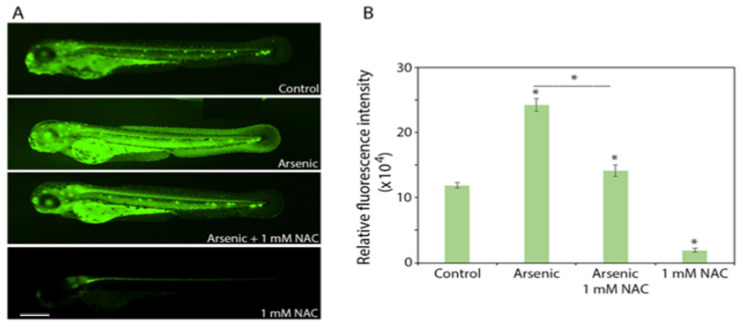
Effect of sodium arsenite and NAC (1 mM), alone or in combination, on cell survival in *hb9-GFP* transgenic zebrafish embryos. Embryos at 5 hpf were exposed (static) to 200 mg/L of sodium arsenite in the presence or absence of 1 mM NAC (*n* = 10/treatment group). After 67 h of exposure (embryo age at the end of the experiment—72 hpf), the embryos were incubated in acridine orange to detect cell death (fluorescent deep green or yellow signal) in vivo. (**A**) Images of whole embryos are shown. (**B**) Quantification of whole-body (excluding the yolk sac and yolk extension) relative fluorescence is presented. Data are presented as mean ± SEM. An * associated with a bar indicates a significant difference (*p* < 0.05) from the control; an * associated with a horizontal line indicates a significant difference (*p* < 0.05) between the treatments indicated. N-acetylcysteine = NAC; sodium arsenite = arsenic. Scale bar: 370 µm.

Sodium arsenite induced extensive apoptosis (as inferred from acridine orange staining) in zebrafish embryos at a concentration of 200 mg/L. Co-treatment with 1 mM NAC significantly reduced sodium arsenite-induced apoptosis (Figure 2A,B). NAC (1 mM) alone abolished the basal physiological level of apoptosis observed in the control embryos (Figure 2A,B).

### 2.3. Although Toxic by Itself, NAC at 2 mM Abolishes Sodium Arsenite-Induced Apoptosis

**Figure 3:**
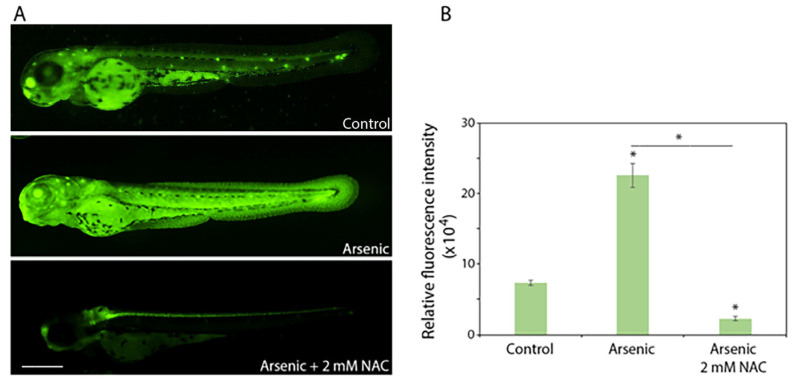
Effect of sodium arsenite and NAC (2 mM), alone or in combination, on cell survival in *hb9-GFP* transgenic zebrafish embryos. Embryos at 5 hpf were exposed (static) to 200 mg/L of sodium arsenite in the presence or absence of 2 mM NAC (*n* = 10/treatment group). After 67 h of exposure (embryo age at the end of the experiment—72 hpf), the embryos were incubated in acridine orange to detect cell death (fluorescent deep green or yellow signal) in vivo. (**A**) Images of whole embryos are shown. (**B**) Quantification of whole-body (excluding the yolk sac and yolk extension) relative fluorescence is presented. Data are presented as mean ± SEM. An * associated with a bar indicates a significant difference (*p* < 0.05) from the control; an * associated with a horizontal line indicates a significant difference (*p* < 0.05) between the treatments indicated. N-acetylcysteine = NAC; sodium arsenite = arsenic. Scale bar: 390 µm.

Sodium arsenite-induced extensive apoptosis (as inferred from acridine orange staining) in zebrafish embryos was completely abolished by 2 mM NAC (Figure 3A,B). In addition, arsenic-induced apoptosis was abolished by NAC at 2 mM (Figure 3A,B). These results indicate that sodium arsenite and NAC have opposing effects at the cellular level that is critical in preventing toxic effects of each other.

### 2.4. Sodium Arsenite or NAC Did Not Alter ROS Production and Protein Oxidation (Protein Carbonylation) in Zebrafish Embryos

**Figure 4:**
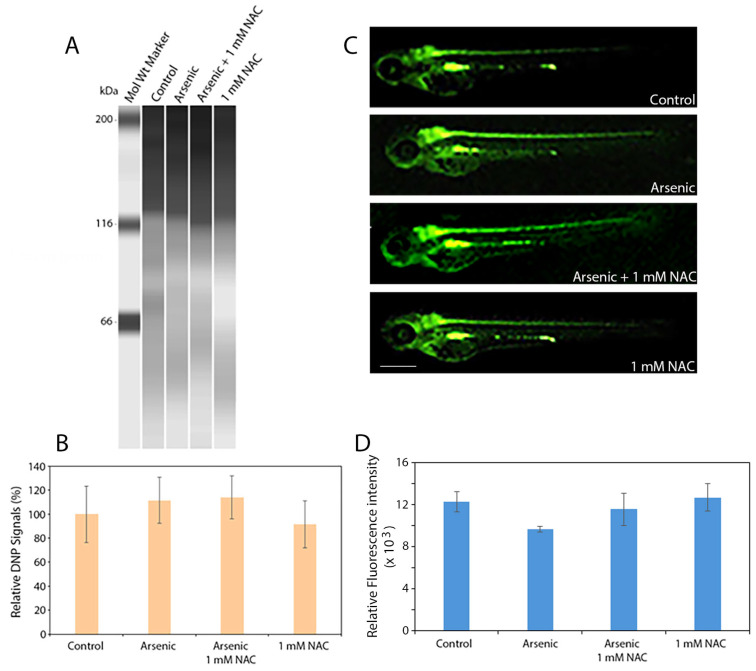
OxyBlot protein oxidation (carbonylation) analysis and ROS detection in *hb9-GFP* transgenic zebrafish embryos (5 hpf) exposed to sodium arsenite (200 mg/L) and 1 mM NAC, alone or in combination. Twenty *hb9-GFP* zebrafish embryos (72 hpf) were sonicated in lysis buffer (RIPA buffer containing 50 mM dithiothreitol) at 15 μL/embryo (10 embryos in 150 μL lysis buffer/treatment group) for 20 s. The lysates were centrifuged at 10,000× *g* for 30 min at 4 °C. Protein samples (equal volume taken from each lysed zebrafish sample) were diluted 1:10 with lysis buffer as described in the manufacture’s protocol, denatured at 95 °C for 5 min, and cooled on ice. (**A**) Five μL from each protein sample (*n* = 6 per treatment group) was processed for OxyBlot analysis. (**B**) Quantification of protein oxidation is shown (*p* = 0.842). (**C**) Relative ROS fluorescence intensities of the experimental groups are shown (*n* = 10); and (**D**) Quantification of the ROS fluorescence intensities of the experimental groups are shown (*p* = 0.263). Scale bar: 380 μm. Data are presented as mean ± SEM. N-acetylcysteine = NAC; sodium arsenite = arsenic.

Protein oxidation (carbonylation) was examined using OxyBlot™ kit combined with Simple-Western analyses. The data showed that neither sodium arsenite nor NAC, alone or in combination, induced protein oxidation (carbonylation) in zebrafish embryos (Figure 4A,B). Due to the use of transgenic zebrafish embryos, in which motor neurons express GFP, a green fluorescent protein, we only assessed ROS-positive signals in the liver and digestive tract that are rapidly developing in the embryos with pronounced cell metabolic activity. ROS detection using H2DCF-DA showed no significant difference between the treatment groups. These results suggest that sodium arsenite or NAC caused no oxidative stress at the concentrations and duration of exposure used.

### 2.5. NAC Does Not Alter Sodium Arsenite-Induced Supernumerary Motor Neuron Development

**Figure 5:**
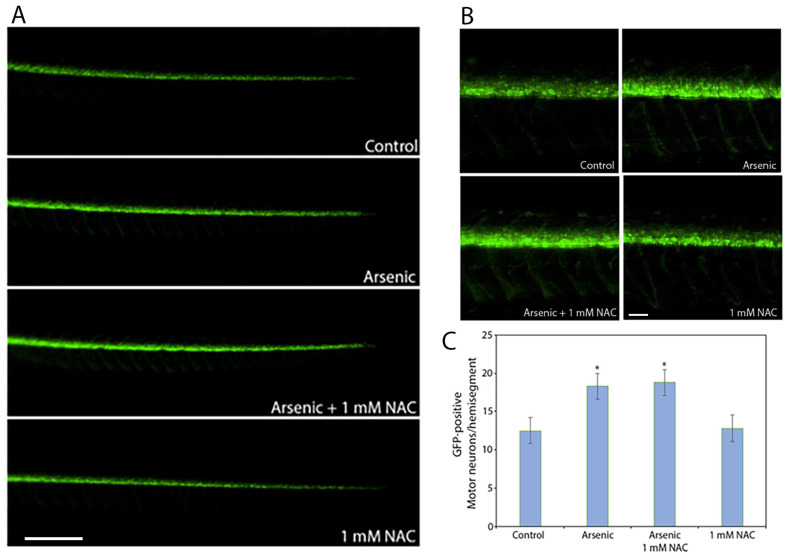
Effect of sodium arsenite and NAC, alone or in combination, on motor neurons in *hb9-GFP* transgenic zebrafish embryos. Embryos (*hb9-GFP*) at 5 hpf were exposed to 200 mg/L of sodium arsenite in the presence or absence of 1 mM NAC (n = 10/treatment group). (**A**) Fluorescent images of spinal cord regions of the embryos are shown. GFP-positive motor neurons are shown in the spinal cord region of control and embryos treated with sodium arsenite in the presence or absence of 1 mM NAC. Scale bar: 350 μm. (**B**) High magnification views of motor neurons distal to the yolk extensions are shown. Motor neurons were counted under the fluorescence microscope. Scale bar: 60 μm. (C) Relative numbers of GFP-positive motor neurons in specific hemisegments are quantified and presented as motor neuron numbers per hemisegment. An * indicates a significant difference (*p* < 0.05) from the control. Data are presented as mean ± SEM. N-acetylcysteine = NAC; sodium arsenite = arsenic.

Our previous study showed that 200 mg/L sodium arsenite promoted supernumerary motor neuron development [42]. Co-treatment of 200 mg/L sodium arsenite and 1 mM NAC, a combination that reduced sodium arsenite-induced apoptosis, did not alter motor neuron development (Figure 5A,B). These data suggest that NAC did not have any effect on the motor neuron development in zebrafish embryos and that the pathway for arsenite-induced supernumerary motor neuron development is distinct from arsenite-induced apoptosis.

### 2.6. Sodium Arsenite-Induced Apoptosis May Not Be Mediated by p53

**Figure 6:**
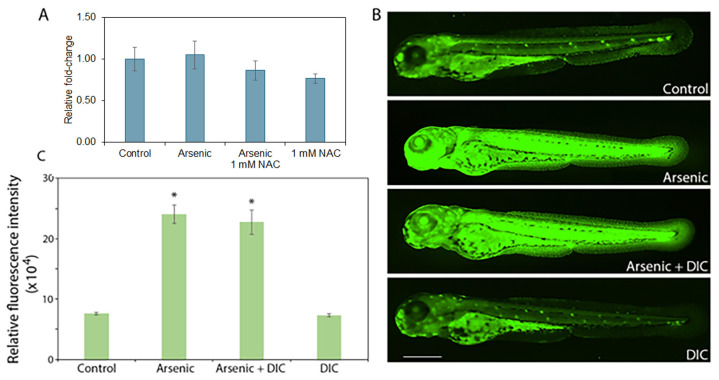
Effect of sodium arsenite and NAC on p53 pathway. (**A**) Sodium arsenite (200 mg/L) or NAC (1 mM), alone or in combination, does not alter *p53* gene expression in *hb9-GFP* transgenic zebrafish embryos (n = 3/treatment group) (*p* = 0.12). For each treatment group, RNA was isolated from 100 pooled embryos; (**B**) The p53 inhibitor dicoumarol (250 nM) does not inhibit sodium arsenite-induced apoptosis in zebrafish embryos (n = 10/treatment group); Scale bar: 430 μm. (**C**) Quantification of whole-body (excluding the yolk sac and yolk extension) relative fluorescence is shown. Data are presented as mean ± SEM. An * indicates a significant difference (*p* < 0.05) from the control. N-acetylcysteine = NAC; dicoumarol = DIC; sodium arsenite = arsenic.

To assess whether sodium arsenite-induced apoptosis (as inferred from acridine orange staining) was mediated by p53 signaling pathway, we first performed qPCR to determine p53 transcript levels. Arsenic or NAC alone or in combination did not alter p53 mRNA expression (Figure 6A). It is known that p53 signaling is mostly induced via post-translational modifications (reviewed in [53]). Since sodium arsenite did not alter p53 levels at the transcriptional level, we used dicoumarol, a chemical that degrades p53 protein [54]. To investigate if p53 activation occurred post-translationally to cause sodium arsenite-induced apoptosis, we tested the effect of dicoumarol. An initial experiment showed that zebrafish embryos could only tolerate 250 nM dicoumarol without causing mortality. Co-treatment with dicoumarol did not inhibit sodium arsenite-induced apoptosis (Figure 6B,C). These data suggested that sodium arsenite-induce apoptosis in zebrafish embryos may not be mediated by p53.

## 3. Discussion

Arsenic is known to induce apoptosis via oxidative stress (reviewed in [55]). For example, human keratinocytes undergo apoptosis upon arsenic exposure [9], and histopathological analysis of lesions from patients with arsenic-induced Bowen’s disease (a form of skin cancer) revealed both abnormal proliferation and apoptosis [56]. In the current study, we used 200 mg/L sodium arsenite, a concentration we have previously shown to induce apoptosis in zebrafish embryos [21,22]. Our prior work demonstrated that this water concentration results in an internal embryonic concentration of 387.8 ± 26.9 pg arsenic per embryo [42]. Given an average embryo body weight of 0.35 mg, this corresponds to approximately 1106 parts per billion (ppb)—substantially higher than the EPA’s maximum contaminant level of 10 ppb for arsenic in drinking water.

While 200 mg/L arsenite concentration, we used in this study, exceeds environmentally relevant levels, it is important to consider cumulative exposure through bioaccumulation over time. Human epidemiological studies support the relevance of such concentrations: increased prostate cancer mortality has been observed in populations with cumulative arsenic exposure of 1000–4999 ppb-years and ≥5000 ppb-years [57]. Additionally, arsenic concentrations in drinking water ranging from 250 to 1140 ppb and 10 to 1752 ppb have been associated with increased prostate cancer mortality [58,59], demonstrating that chronic exposure to elevated arsenic levels poses significant health risks. Arsenic induces apoptosis in both cancerous and healthy cells, including myoblasts, pancreatic cells, brain cells [33,43,44,45], keratinocytes [9], and T-lymphocytes [10]. One of the mechanisms involves arsenic-induced oxidative stress [46]. The proapoptotic effect of arsenic has been associated with cancerous skin conditions [12]. Our study demonstrates that acute arsenite exposure (67 h) induced apoptosis in zebrafish embryos, consistent with data from mammalian cells.

Arsenic-induced apoptosis operates through the activation or inactivation of multiple signaling cascades [13], including oxidative stress pathways and the tumor suppressor protein p53 [14,15]. In the current study, we investigated the dose-dependent effects of NAC in the presence of inorganic arsenic (sodium arsenite) in zebrafish embryos. We hypothesized that NAC, as an antioxidant dietary supplement, would prevent sodium arsenite-induced apoptosis. This hypothesis was based on our earlier finding that ALCAR, another antioxidant dietary supplement [60], prevented sodium arsenite-induced apoptosis in zebrafish embryos [21]. Our co-treatment experiments revealed that 1 mM NAC alone did not affect zebrafish development or survival, whereas 2 mM NAC alone induced 100% mortality. These findings are consistent with several reports of NAC-induced apoptosis in in vitro studies. For instance, NAC induced apoptosis in human leukemia cells and mouse cortical neurons through oxidative stress [61,62] and caused apoptosis in rat H9c2 myocardium cells via endoplasmic reticulum stress [63]. Although uncommon, antioxidant toxicity has been documented, particularly at high doses [64,65]. NAC has been shown to increase oxidative stress in mouse liver, causing microvesicular steatosis by depleting glutathione levels [66]. Furthermore, a case study reported that NAC overdose (100 g instead of 10 g administered within a short timeframe) led to acute renal failure, thrombocytopenia, and hemolysis, ultimately resulting in patient death—underscoring the importance of careful NAC dosing [67]. In the current study, 2 mM NAC administered over 67 h likely constituted an overdose for zebrafish embryos. Notably, the improved survival of embryos co-treated with both sodium arsenite and 2 mM NAC highlights the counteracting effects of these two chemicals.

Multiple studies have demonstrated that various chemicals provide protection against arsenic toxicity. In mice, betaine protects against sodium arsenite-induced cardiotoxicity through its antioxidative properties [68]. Similarly, melatonin reduced arsenic-induced oxidative stress and apoptosis in zebrafish embryos, preventing cardiotoxicity [69]. Several in vitro studies have shown beneficial effects of NAC on cell survival against arsenic-induced toxicity, including in human leukemia (HL-60) cells [70], mouse Oli-neu oligodendrocyte cells [71], and mouse 3T3 embryonic fibroblast cells [72]. Additionally, NAC prevented arsenic-induced apoptosis in a zebrafish liver cell line in vitro [36]. One in vivo study examined NAC’s protective effects in zebrafish embryos, but only assessed a restricted cell population in the head region where arsenic-induced apoptosis was ameliorated by NAC [37]. However, the effect of NAC on arsenic-induced apoptosis across all cell types in whole zebrafish embryos in vivo had not been previously demonstrated. The current study addresses this gap by showing that 2 mM NAC completely abolished sodium arsenite-induced apoptosis throughout zebrafish embryos, as evidenced by acridine orange staining. These findings suggest that NAC’s antioxidative properties provide systemic protection against arsenic-induced cell death in vivo.

Our previous study demonstrated that ALCAR counteracted arsenic-induced apoptosis but had no effect on motor neuron development [21]. Similarly, in the current study, NAC did not affect sodium arsenite-induced supernumerary motor neuron development. These findings suggest that arsenic-induced supernumerary motor neuron development and apoptosis occur through distinct pathways in zebrafish embryos.

We previously showed that arsenic induces supernumerary motor neurons in zebrafish embryos through Sonic hedgehog (Shh) pathway activation [42]. While low-dose γ-ray irradiation in zebrafish embryos has been shown to both induce apoptosis and increase Shh at the mRNA and protein levels [73], a direct mechanistic link between Shh and apoptosis was not established in that study. Notably, Shh is known to inhibit apoptosis in zebrafish retina and human tumor cells [74,75]. Given this anti-apoptotic role of Shh and our previous finding that arsenic activates Shh signaling [42], it is unlikely that arsenic-induced Shh activation plays a role in arsenic-induced apoptosis. Instead, these data support the conclusion that arsenic affects motor neuron development and apoptosis through independent mechanisms. However, further studies are warranted to determine the role of Shh in arsenic-induced apoptosis.

Oxidative stress occurs when there is excessive production of reactive oxygen species (ROS) (reviewed in [76]). In mammalian cells, inorganic arsenic toxicity is partially mediated by oxidative stress [17]. For example, salvianolic acid A, an antioxidant, has been shown to prevent arsenic trioxide-induced endoplasmic reticulum stress-driven apoptosis [77]. Protein carbonylation—an irreversible modification of amino acids such as lysine, arginine, and proline—is a hallmark of severe oxidative stress [78]. Arsenic is a known inducer of both oxidative stress [46] and protein carbonylation [79,80,81,82]. However, the relationship between arsenic exposure and protein carbonylation appears to be dose- and duration-dependent. Low-dose, short-term arsenic exposure (up to 72 h) induced proteotoxic stress without causing detectable protein carbonylation in human and mouse cell lines [83]. Similarly, arsenic exposure in humans did not induce protein carbonylation as assessed in urine and blood samples [84]. The current study also showed no effect of arsenic on protein carbonylation, either in the presence or absence of NAC, suggesting that 67 h exposure was insufficient to cause this irreversible modification in zebrafish embryos. Additionally, NAC alone had no effect on protein carbonylation, consistent with findings in rat heart tissue [85]. These results align with the understanding that protein carbonylation does not always occur upon oxidative stress, as cells possess protective mechanisms that prevent this modification. While other oxidative modifications may occur, carbonylation is not easily induced and typically requires more severe or prolonged oxidative conditions [86].

The relationship between arsenic exposure and ROS production appears to be complex and context-dependent. In zebrafish embryos exposed to 120 mg/L sodium arsenite for 5 days—two days longer than our current study—ROS levels were significantly increased, and selenium prevented this elevation [87]. However, contradictory results have been reported in other systems. For instance, low-level arsenite exposure (2 μM or 259.82 μg/L) did not induce ROS production in NIH3T3 mouse fibroblast cells [83]. Chronic sodium arsenite treatment (8–24 weeks) either increased or decreased ROS levels depending on cell type and exposure duration [88,89]. Additionally, ROS was found to be unimportant in arsenic trioxide-induced apoptosis in a multiple myeloma cell line [90]. In contrast, studies using human colorectal cells (HCT 116) and human embryonic kidney cells (HEK293ET) demonstrated that arsenite induced increased ROS levels, which were significantly reduced by 2.5 mM and 10 mM NAC, respectively [91,92]. Although the current study shows no detectable induction of ROS in zebrafish embryos, our data do not rule out sodium arsenite-induced oxidative stress. Oxidative stress could still be occurring through alternative mechanisms—such as generation of other reactive species (e.g., reactive nitrogen species), depletion of antioxidants, or time-dependent effects—without producing detectable protein carbonylation, which represents only one of many downstream consequences of oxidative stress.

Apoptosis is induced by p53, a well-known tumor suppressor [14,93]. Sodium arsenite has been shown to induce p53 expression in cultured mammalian cells [94] and primary human keratinocytes [95]. In adult zebrafish livers, sodium arsenite significantly increased *p53* gene expression after 28 days of exposure [96]. However, the current study showed no change in *p53* gene expression in zebrafish embryos, and NAC similarly had no effect on *p53* gene expression. The absence of changes in *p53* gene expression does not necessarily exclude p53’s involvement in arsenic-induced apoptosis, as p53-mediated apoptosis is primarily regulated through posttranslational modifications rather than transcriptional changes [97]. To investigate whether p53 protein plays a role in sodium arsenite-induced apoptosis, we used dicoumarol, a chemical that degrades p53 protein. Our results showed that dicoumarol co-treatment did not prevent sodium arsenite-induced apoptosis in zebrafish embryos, indicating that apoptosis occurred independently of p53 signaling. These findings suggest that arsenic induces apoptosis through alternative pathways in zebrafish embryos, at least under the exposure conditions and developmental timepoint examined in this study.

In conclusion, the present study demonstrates the efficacy of NAC against sodium arsenite-induced apoptosis in zebrafish embryos. NAC prevented sodium arsenite-induced apoptosis in a dose-dependent manner. Notably, this protective effect did not involve alterations in ROS production or protein carbonylation, two hallmarks of oxidative stress. Furthermore, neither NAC nor sodium arsenite altered the expression of the pro-apoptotic gene *p53*, and dicoumarol, which degrades p53 protein, did not prevent sodium arsenite-induced apoptosis. These findings collectively indicate that arsenic-induced apoptosis occurs through a p53-independent mechanism in zebrafish embryos.

Future studies will focus on specific detection of apoptosis using the TUNEL (Terminal deoxynucleotidyl transferase dUTP nick end labeling) and caspase 3 activation assays since acridine orange staining may not be able to distinguish between apoptotic and necrotic cells, although a green filter was used in our study to detect only apoptosis. Several alternative pathways have been implicated in arsenic-induced apoptosis, including mitochondria-mediated pathways (mitochondrial membrane potential loss, caspase-9/3 activation, and cytochrome *c* release), endoplasmic reticulum stress pathways, and mitogen-activated protein kinase (MAPK) signaling pathways (reviewed in [98]). Future studies exploring these pathways are warranted to elucidate the precise mechanisms underlying NAC’s protective effects against sodium arsenite-induced apoptosis in zebrafish embryos. Such investigations would enhance our understanding of arsenic’s adverse effects in human populations and potentially reveal therapeutic avenues for ameliorating arsenic toxicity.

## 4. Materials and Methods

### 4.1. Animals

Adult *GFP-hb9* transgenic zebrafish (*Danio rerio*, AB strain) [78] were purchased from the Zebrafish International Resource Center (www.zirc.org, accessed on 3 February 2026) (ZIRC, Eugene, OR, USA). The care and maintenance of zebrafish were based on the guidelines in the National Institutes of Health (NIH) Guide for the Care and Use of Laboratory Animals (https://www.ncbi.nlm.nih.gov/books/NBK54050/, accessed on 3 February 2026). All animal care and handling procedures were approved by the National Center for Toxicological Research, U.S. Food and Drug Administration’s Institutional Animal Care and Use Committee. Adult zebrafish were kept in 1 L tanks (Aquatic Habitats, Apopka, FL, USA) in the zebrafish facility at 28.5 °C in recirculating reverse osmosis buffered water (pH 7.5, conductivity 800 μs, and dissolved oxygen level set at 7 ppm). Adult zebrafish were fed daily with live brine shrimp (AquaCave, Inc., Lake Forest, IL, USA) and Zeigler dried flake food (Zeiglers, Gardeners, PA, USA). The light: dark cycle in the zebrafish room was maintained at 14:10 h. Breeding of zebrafish was performed using in-system breeding tanks, in which adult zebrafish were kept in the tanks with partitions separating the males and females. The partitions were removed the following morning at the onset of light to stimulate spawning. Fertilized eggs were collected in Petri dishes after repeated washing with recirculating reverse osmosis buffered (with sodium bicarbonate) water (pH 7.2–7.5). The eggs were kept in an incubator at 28.5 °C for all experiments.

### 4.2. Reagents

Sodium arsenite (NaAsO_2_) (CAS No.: 7784-46-5; purity ≥ 90%), 2′,7′-dichlorodihydrofluorescein diacetate (CAS No.: 4091-99-0; purity ≥ 97%), and N-acetylcysteine (CAS No.: 616-91-1; purity ≥ 99%), acridine orange (CAS No.: 65-61-2; purity ≥ 95%) were purchased from Sigma-Aldrich (St. Louis, MO, USA). The identity and purity of sodium arsenite was confirmed by inductively coupled plasma mass spectrometry [42]. The source of all other chemicals used in this study was Sigma-Aldrich unless otherwise indicated. Sodium arsenite and NAC solutions were made fresh with embryo medium that contained 15 mM NaCl, 0.5 mM KCl, 1 mM MgSO_4_, 0.15 mM KH_2_PO_4_, 0.05 mM Na_2_HPO_4_, and 0.7 mM NaHCO_3_ [52].

### 4.3. Treatment of Zebrafish Embryos with Sodium Arsenite and N-Acetylcysteine

*hb9-GFP* transgenic zebrafish embryos were used in this study. We have used this strain previously to investigate the effect of arsenic [42]. For treatment with sodium arsenite (200 mg/L) and NAC (1 or 2 mM), 5 hpf embryos were used. This concentration of sodium arsenite was chosen based on our previous study that showed that there were no changes in overall development and morphology [42]. Exposure to sodium arsenite was conducted in the presence or absence of 1 or 2 mM NAC. We used these two concentrations of NAC based on our previous study related to developmental toxicity in zebrafish embryos [99]. For each treatment, 10–12 embryos were placed in each well of six-well plates containing 5 mL embryo medium. At 72 hpf of age (67 h exposure of 5 hpf embryos), embryos were assessed to detect mortality, apoptosis, protein carbonylation (oxidative stress), and motor neuron development. Each experiment was repeated at least three separate times on different days using eggs from different breeders (n = 10/treatment group). The data presented are representative of these replications.

### 4.4. Acridine Orange (AO) Staining of Live Zebrafish Larvae for Detection of Apoptosis

To detect apoptosis, we stained the embryos with the vital dye, acridine orange (AO; acridinium chloride hemi-[zinc chloride]) [100,101] as described in our previous study [21,22,101]. AO staining infers apoptosis by acting as a membrane-permeable dye that binds to nucleic acids. It indicates apoptotic cells through green fluorescence for DNA fragmentation, and red-orange fluorescence for cytoplasmic changes such as lysosomal degradation, markers of necrotic cells. We used the green channel to image the embryos to detect apoptotic cells. Embryos, at 72 hpf (67 h post exposure to the chemicals), were incubated for 1 h with 5 μg/mL of AO in embryo water. Embryos were washed with embryo water three times and were imaged using the green filter of a Nikon SMZ18 microscope and DS-Ri2 Color CMOS camera (Nikon, Tokyo, Japan). Integrated fluorescence intensities from the images (with the use of color threshold) of the body region (excluding the yolk sac and yolk extension) of embryos from each experimental group (*n* = 10) were quantified using ImageJ (v1.53K) image analysis software (U.S. National Institutes of Health, Bethesda, Maryland, USA). Yolk sac and yolk extension were excluded because of unpredictable autofluorescence emanating from those parts of the embryos, which is a common occurrence. The body region of each embryo image was selected using the freehand selections tool from ImageJ menu. All the experiments have been replicated at least three times on different days using eggs from different breeders (*n* = 10/treatment group). The data presented are representative of these replications. Relative fluorescence data are presented as mean ± standard error of the mean (SEM). Significant differences (*p* < 0.05) between the treatment groups were determined by one-way ANOVA using Holm–Sidak pairwise multiple comparison post hoc analysis (SigmaPlot 14.5).

### 4.5. Assessment of Motor Neuron Development in Transgenic Zebrafish Embryos

Zebrafish *hb9-GFP* transgenic embryos [102] were used to assess motor neuron development. The hb9 transcription factor is expressed in the developing motor neurons of zebrafish [103,104] and mammals [105]. In these transgenic zebrafish embryos, motor neurons express green fluorescent protein (GFP) under the control of a promoter containing specific regulatory elements of the zebrafish hb9 gene [102,106]. After treating with sodium arsenite in the presence or absence of NAC, images of the transgenic larvae were acquired using a Nikon SMZ18 microscope and DS-Ri2 Color CMOS camera. In the embryos, GFP-expressing spinal motor neurons in the region that constituted three consecutive hemisegments, starting from the distal end of the yolk extension, were visually counted under a fluorescent microscope as described in our previous studies (*n* = 10/treatment group) [42,101,107]. The average number of motor neurons from the three hemisegments was considered as the motor neurons per hemisegment. Visual counting is necessary because microscope camera can not capture all the motor neurons on the other side of the hemisegment overlapped by the motor neurons facing the microscope camera. The experiments have been repeated at least three times on different days using eggs from different breeders. Data from a representative experiment are presented. The values were averaged to obtain the number of neurons/hemisegment. The experiment was repeated three times to ensure reproducibility. Data are presented as mean ± SEM. Significant differences (*p* < 0.05) between the treatment groups were determined by one-way ANOVA using Holm–Sidak pairwise multiple comparison post hoc analysis (SigmaPlot 14.5).

### 4.6. OxyBlot Assay for the Measurements of Protein Oxidation (Protein Carbonylation)

Protein oxidation was examined using OxyBlot™ kit (Millipore Sigma, Rockville, MD, USA) combined with Jess™ Simple-Western analyses (ProteinSimple, San Jose, CA, USA). Embryos (72 hpf) were sonicated in lysis buffer (RIPA buffer containing 50 mM dithiothreitol) at 15 μL/embryo (10 embryos in 150 μL lysis buffer/treatment group) for 20 s (n = 6/treatment group). The lysates were centrifuged at 10,000× *g* for 30 min at 4 °C. Protein samples (equal volume taken from each lysed zebrafish sample) were diluted 1:10 with lysis buffer as described in the manufacture’s protocol, denatured at 95 °C for 5 min, and cooled on ice. Five mL from each protein sample was mixed with 5 mL 12% sodium dodecyl sulfate (SDS) and then 10 mL 2,4-dinitrophenylhydrazine (DNPH) solution was added. After incubation at room temperature for 15 min, 7.5 mL neutralization solution was added to each sample to quench the chemical reactions. The protein samples were then mixed with fluorescent 5× Master Mix (4:1 *v*/*v*, ProteinSimple), loaded into capillaries, separated, immobilized, incubated with rabbit anti-dinitrophenyl (DNP) antibody (1:50, Millipore) for 30 min, washed, and then incubated with horse radish peroxidase-conjugated anti-rabbit antibodies for 30 min. After washing, the capillaries were incubated with the luminol-S/peroxide substrates, and chemiluminescence signals were captured using a charge-coupled device (CCD) camera. After the chemiluminescence signals of the DNP-derivatized proteins (carbonylated proteins) in each capillary were captured, the chemiluminescence signals were stripped using a RePlex™ kit (ProteinSimple). Total proteins in each capillary were then determined using Simple-Western Total Protein Detection Module (ProteinSimple), which is a chemiluminescence-based total protein assay kit. The chemiluminescence signals of the DNP-derivatized proteins or carbonylated proteins and the total protein in each capillary were quantified using Compass software version 6.3.0 (ProteinSimple). The signal intensity of the DNP-derivatized proteins in each capillary were normalized automatically by the Compass software based on the signals of the total proteins in the capillary. The normalized signal intensity represents the relative abundance of oxidized proteins.

### 4.7. Detection of Reactive Oxygen Species (ROS) in Zebrafish Embryos

ROS in zebrafish embryos were measured by fluorescence microscopy using the cell membrane-permeable compound, 2′,7′-di-chlorodihydrofluorescein diacetate (H2DCF-DA) fluorescent dye, a permanent ROS marker [31], essentially following a method used for zebrafish embryos [32] as in our previous studies [108,109]. H2DCF-DA is deacetylated by cellular esterases to non-fluorescent 2′,7′-dichlorofluorescein (DCFH). In response to cellular ROS production, DCFH is converted to the green fluorescent di-chlorofluorescein (DCF), the fluorescent intensity of which can be visualized and quantified using fluorescence microscopy. In brief, zebrafish embryos were stained with H2DCF-DA (50 mM) for 60 min in dark at 28.1 °C. The fluorescence of the whole embryo was visualized using a Nikon SMZ18 microscope and DS-Ri2 Color CMOS camera through the green channel and images were acquired. Fluorescence intensities of the digestive tract and liver (where ROS is highly expressed [108,109,110,111], possibly due to the fast developing digestive system, where cell metabolic activity must be high with pronounced mitochondrial activity at the time, relative to other parts of the body) from each experimental group (n = 10) were quantified using ImageJ image analysis software. The experiments have been repeated at least three times on different days using eggs from different breeders. Data from a representative experiment are presented. Data for each treatment group (n = 10) were used to calculate the mean ± SEM. Significant differences (*p* < 0.05) between the treatment groups were determined by one-way ANOVA using Holm–Sidak pairwise multiple comparison post hoc analysis (SigmaPlot 14.5).

### 4.8. RNA Extraction and cDNA Synthesis

RNA extraction was performed as described in our previous studies [112,113]. Total RNA (pooled from 100 embryos [72 hpf]/treatment group) from three independent samples for each experimental group was extracted using Trizol reagent (Invitrogen, Carlsbad, CA, USA) followed by isolation using an RNeasy kit (Qiagen, Germantown, MD, USA). The RNA samples were treated with DNase I (Gene Copoeia, Rockville, MD, USA) followed by heat inactivation in the presence of EDTA. The RNA concentration and purity were assessed spectrophotometrically using a NanoDrop 2000 (NanoDrop Technology, Wilmington, DE, USA) and the RNA integrity was verified using a 2100 Bioanalyzer (Agilent Technologies, Santa Clara, CA, USA). The absorption 260/280 ratio of all 12 RNA samples was 2.1 ± 0.01 and the RNA integrity number was 9.5 ± 0.3 (mean ± standard deviation). The first strand complementary DNA (cDNA) was synthesized from total RNA (4.5 μg; 20 μL final reaction volume) with oligo (dT) priming using SuperScript III reverse transcriptase (ThermoFisher, Waltham, MA, USA) according to the manufacturer’s instructions.

### 4.9. Quantitative Polymerase Chain Reaction (qPCR)

Quantitative polymerase chain reaction (qPCR) analyses were performed using a CFX96 C1000 PCR system (Bio-Rad, Hercules, CA, USA). The reaction mixture in each well (25 μL final volume) contained 1 × SsoFast EvaGreen Supermix (Bio-Rad) and 250 nL cDNA. The PCR amplification cycling parameters were 50 °C × 2 min, 95 °C × 10 min, then 40 cycles of 95 °C × 15 s, 60 °C × 1 min. Then a melting temperature-determining dissociation step followed at 95 °C × 15 s, 60 °C × 15 s, and 95 °C × 15 s. The specificity of amplification reactions was confirmed by melting curves that consistently showed a single peak. Genes that showed expression threshold cycle (Ct) values > 35 were excluded. The primers for the housekeeping gene (*gapdh*) were forward 5′-GATACACGGAGCACCAGGTT-3′ and reverse 5′-GCCATCAGGTCACATACACG-3′. In the qPCR assay, a no-template (cDNA) control for reagent contamination and a no-reverse-transcriptase control to detect genomic DNA contamination were included. The primers for *p53* were forward 5′-CTCTCCCACCAACATCCACT-3′ and reverse 5′-GTTATCTCCATCCGGGGTTC-3′. Data from the qPCR for *p53* expression were normalized against the expression of *gapdh* by calculating the average delta cycle threshold (ΔCt) for each gene on the plate. Three samples (n = 3) for each experimental group were used. The relative levels of gene expression (sodium arsenite-treated versus control) were calculated using the comparative delta–delta-Ct method [114].

### 4.10. Treatment of Zebrafish Embryos with Sodium Arsenite in the Presence or Absence of Dicoumarol (DIC), a p53 Inhibitor

The NAD(P)H: quinone oxidoreductase 1 (NQO1) inhibitor dicoumarol acts as an inhibitor of the p53 signaling by degrading p53 protein through proteasomal degradation, which results in the inhibition of p53-mediated apoptosis [49]. A dicoumarol (Cat # 287897; Sigma-Aldrich) stock solution was made in dimethyl sulfoxide at 20 mM. *hb-GFP* transgenic zebrafish embryos were used in this study. For treatment with sodium arsenite (200 mg/L) and dicoumarol (250 nM), 5 hpf *hb9-GFP* transgenic zebrafish embryos were used [38]. Exposure to sodium arsenite was conducted in the presence or absence of 250 nM dicoumarol since doses more than 250 nM caused teratogenicity and mortality (our unpublished data). For each treatment, 10–12 embryos were placed in each well of six-well plates containing 5 mL embryo medium. At 72 hpf of age (67 h exposure of 5 hpf embryos), embryos were collected for assessment of apoptosis (n = 10/treatment group). The experiments have been repeated at least three times on different days using eggs from different breeders. Data from a representative experiment are presented.

### 4.11. Statistical Analysis

Statistical significance of embryo survival, AO fluorescence to detect apoptosis, OxyBlot protein oxidation values, motor neuron numbers, and gene expression (fold change) was calculated using one-way ANOVA (SigmaPlot 14.5). Values are presented in figures as mean ± SEM. Changes were considered significant at *p* ≤ 0.05 (indicated by *).

## Data Availability

The original contributions presented in this study are included in the article. Further inquiries can be directed to the corresponding author.
